# HIF-1α/BNIP3L induced cognitive deficits in a mouse model of sepsis-associated encephalopathy

**DOI:** 10.3389/fimmu.2022.1095427

**Published:** 2022-12-07

**Authors:** Lina Zhao, Yu Song, Ying Zhang, Haiying Liu, Yuehao Shen, Yan Fan, Yun Li, Keliang Xie

**Affiliations:** ^1^ Department of Critical Care Medicine, Tianjin Medical University General Hospital, Tianjin, China; ^2^ Department of Anesthesiology, Tianjin Medical University General Hospital, Tianjin, China; ^3^ Tianjin Research Institute of Anesthesiology, Tianjin, China

**Keywords:** sepsis associated encephalopathy, HIF-1α, BNIP3L, sepsis, mechanism

## Abstract

**Objective:**

Sepsis Associated Encephalopathy (SAE) is a common complication in critically ill patients and perioperative period, but its pathogenesis is still unclear. This study aimed to explore the effect of the HIF-1α (hypoxia-inducible factor-1α)/BNIP3L (Bcl-2/adenovirus E1B 19-kDa interaction protein) signaling pathway on SAE.

**Methods:**

C57BL/6J male mice were divided into four groups, using a random number table method: control group, sham group, sepsis group, sepsis+HIF-1α activity inhibitor (echinomycin) group. Sepsis was induced by cecal ligation and puncture (CLP). At 24 h after surgery, brain tissue was sampled. HE was staining to observe changes in the hippocampus structure. Fluoroscopy observes changes in mitochondrial structure. Western blot, QT-PCR, and immunofluorescence were used to assess the amount of expression of HIF-1α and BNIP3L in the hippocampus and mitochondrion of hippocampus neurons. Observation of neuronal apoptosis by TUNEL staining. Seven days after surgery, mice were tested in a Morris water maze test to assess cognitive function after CLP.

**Results:**

Our results show that CLP-induced hippocampus-dependent cognitive deficits were accompanied with increased HIF 1a and decreased BNIP3L, increased protein levels of TNF-α, IL-6, and IL-β, and damage to mitochondrial structures and neuronal apoptosis in the hippocampus. In addition, administration of echinomycin rescues cognitive deficits, ameliorates HIF-1α and BNIP3L-mediated neuronal pyroptosis and damaged mitochondrial structures, and decreases the expression of TNF-α and IL-6 in the hippocampus.

**Conclusions:**

HIF-1α and the BNIP3L promote mitochondrial damage, and neuronal apoptosis and the expression of inflammatory factors may be the mechanism of SAE in critically ill patients and perioperative period

## Introduction

Sepsis-associated encephalopathy (SAE) is an acute diffuse dysfunction of the brain caused by infection (1). Clinical studies had found that in about 70% of sepsis patients with cognitive dysfunction after sepsis progresses to encephalopathy in in critically ill patients and perioperative period (2), the patient’s hospital stay is prolonged, the cost of hospitalization increases, and the mortality rate is about 60% (3). The pathogenesis of SAE includes an inflammatory response, astrocytes and microglia activation, mitochondrial dysfunction, and the impaired blood-brain barrier (4–6). SAE has a high incidence and poor prognosis, but its pathogenesis remains unclear. Clinicians have no effective treatment regimen. Therefore, it is important to explore the pathogenesis of SAE, prevent the occurrence of SAE, reduce the incidence of SAE, and improve the prognosis of patients with sepsis.

TheHIF-1α/BNIP3L pathway regulates cell survival, apoptosis, autophagy, and other processes important signaling pathways. Wu C et al. found that inhibition ofHIF-1α/BNIP3L expression improves early brain injury after experimental subarachnoid hemorrhage in rats (7). Barteczek P et al. found that neuronal HIF-1α and HIF-2α deficiency improves neuronal survival and sensorimotor function in the early acute phase after ischemic stroke (8). Yan Jun et al. found that repeated administration of ketamine can induce hippocampus neurodegeneration and long-term cognitive impairment *via* the ROS/HIF-1α pathway in developing rats (9). The study by Lu N et al. confirmed that increased BNIP3L expression could improve autophagy and apoptosis (10). The above studies show that HIF-1α and BNIP3L play an important role in many brain diseases and cognitive dysfunction. Therefore, we speculate that HIF1-a and BNIP3L may play an important role in cognitive dysfunction caused by SAE.

This study hypothesizes thatHIF-1α/BNIP3L promotes SAE cognitive dysfunction and intervenes in their expression, which can improve SAE mice’s cognitive function. To prove this hypothesis, we will explore it from the SAE mice’s behavior and the cellular level.

## Materials and methods

### Animals

The experimental procedures and the animal use and care protocols were approved by the Committee on Ethical Use of Animals of Tianjin Medical University General Hospital. All experimental procedures in this study were performed according to the Guidelines for the Care and Use of Laboratory Animals from the National Institutes of Health, USA. Two hundred and thirty-two C57BL/6 male mice aged 6 to 8 weeks were purchased from the Animal Center of Laboratory Animal Center of the Military Medical Science Academy (Beijing, China) and randomized to 4 groups: control group (n=52), sham group (n=54), sepsis group (n=64) and sepsis+HIF-1α activity inhibitor (echinomycin) group (n=64). The control group did not receive surgery. Mice in the sham group received laparotomy but were without the classical cecal ligation and puncture (CLP). Mice in the sepsis group had CLP. Experiments began after mice had acclimated to the environment for two weeks. Mice were housed in groups of four individuals per cage with a 12:12 h light: dark cycle at a temperature of 20–22°C with food and water available adlibitum.

### Sepsis model

The classical CLP model was applied in this experiment (11). The mice were anesthetized using 2% sodium pentobarbital (50 mg/kg). Under aseptic conditions, the cecum was isolated, and a single puncture with a 21 G needle through the cecum was made between the ligation site and the tip of the cecum. A small number of feces was extruded from the puncture point. The bowel was returned to the abdomen, and the incision was closed with a sterile 6-0 silk suture. For mice that received surgeries, 50 ml/kg of normal saline was injected subcutaneously. Mice subjected to the sham operation had the cecum exposed the same way as for CLP, but it was neither ligated nor punctured.

### Drug administrations

Echinomycin (cat. no. ab 144247; Abcam, Cambridge, MA, USA), an effective and small molecule inhibitor of cell permeability hypoxia inducible factor-1 (HIF-1) DNA binding activity, was used in the experiment with 50μg/kg intraperitoneal injection 2 h after surgery (12).

### Experimental procedures

**Figure 1:**
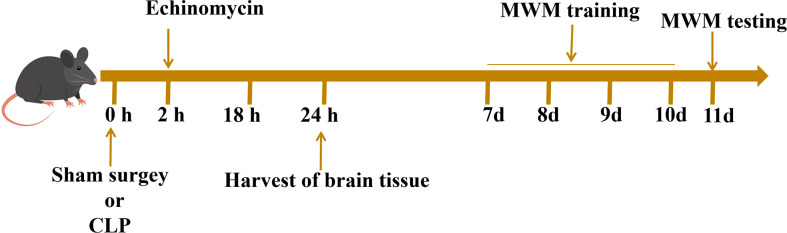
Experimental procedures.Two hours after the operation, the mice in the inhibitor group were injected with echinomycin. The hippocampus tissue of mice were extracted 24 hours after the operation for subsequent analysis. Surviving mice were subjected to behavioral experiments on the 7th day after the operation.

The mice were divided into four groups: the control group, the sham group, the CLP group, and the (sepsis + echinomycin) group, (sepsis + echinomycin) group. The (sepsis+ echinomycin) group was injected intraperitoneally at 2 h after LCP, the mice were sacrificed, and hippocampus were obtained from the different groups at 24 h after CLP. The different groups of mice were given the Morris water maze (MWM) test from the 7th day to the 10th day after the sham or CLP operation (**Figure 1**).

### HE staining

The hippocampus of mice was transversely cut at a 2 mm thickness, immediately fixed in 4% paraformaldehyde, and embedded in paraffin. Sections of 3 μm in thickness were affixed to slides, deparaffinized, and stained with H&E to evaluate morphological changes in the hippocampus.

### Western blotting analysis

Each group of six mice was used for western blotting of the hippocampus, and each group of three mice was used for western blotting of the mitochondrial. The hippocampus and the mitochondrial protein of hippocampus were extracted, the expression level ofHIF-1α, BNIP3L protein of hippocampus and mitochondrial were detected by western blot method (n=9 for each group), specimens were homogenized in precooled radioimmunoprecipitation assay buffer, add high-efficiency RIPA lysate, homogenize, centrifuge at 4 °C, take the supernatant. Proteins were transferred from gel to PVDF membrane and blocked with 5% nonfat milk powder for 1H at room temperature. Add HIF-1α (dilution ratio average 1:1000, cat. no. ab 179483; Abcam, Cambridge, MA, USA) and BNIP3L primary antibody (dilution ratio average 1:1000, cat. no. ab ab 155010; Abcam, Cambridge, MA, USA), shaker at 4°C overnight on the membrane. Washed three times with TBST buffer, added HRP labeled secondary antibody (dilution ratio: 1:5000), incubated at room temperature for 1 h, developed and analyzed by Image J v1.8.0 software.

### Quantitative PCR

Each group of six mice was used for Quantitative PCR. Total RNA from the cells was extracted by using a Trizol reagent (Invitrogen, Waltham, MA, USA) according to the manufacturer manual. Complementary DNA (cDNA) was synthesized by using oligo (dT) primer and PrimeScript™ RT Reagent Kit (Takara, Shiga, Japan). Quantitative PCR (qPCR) was performed to amplify the cDNA using the SYBR Premix Ex Tag Kit (Takara, Shiga, Japan) and an ABI 7500 Sequencing Detection System (Applied Biosystems, Foster City, CA, USA). The gene primers were as follows: BNIP3L, forward 5’- ATGAACAGCAGCAATGGCAATG - 3’, reverse 5’-TGGATGGAAGACGAGGAAGGAA-3’;HIF-1α, forward 5’- GCCTTAACCTGTCTGCCACTT - 3’, reverse 5’- TTCGCTTCCTCTGAGCATTCTG - 3’.B-action internal parameter: forward 5’- GTACTCTGTGTGGATCGGTGG -3’, reverse 5’- GCAGCTCAGTAACAGTCCG -3’.The relative gene expressions were calculated in accordance with the ΔΔCt method. Relative mRNA levels were expressed by the values of 2−ΔΔCt as described previously (13).

### Immunofluorescence staining

Mice (6/group) were anesthetized and sacrificed following at 24 h after surgery. The whole brain tissues were extracted immediately and cut sagittally into hemispheres. Right hemispheres were fixed in ice-cold 4% PFA overnight at 4 °C and were then equilibrated in 30% sucrose. Next, 25-μm sagittal slices in the hippocampus were obtained by a freezing microtome. Sections were washed in cold PBS three times, blocked with 5% goat serum for 1 h, and incubated with HIF-1α antibody and BNIP3L antibody overnight at 4 °C.

### TUNEL assay for cell apoptosis

The hippocampus neurons cells were fixed with paraformaldehyde for 30 minutes and treated with a permeabilizing solution (0.1% Triton X-100) at 4°C for 2 min and incubated in a TUNEL reaction mixture for 60 min at 37°C in the dark. The hippocampus neuron cells were counterstained with 4′,6-diamidino-2-phenylindole (DAPI).

### Transmission electron microscopy

Twenty-four hours after CLP (5/group), the mitochondrial morphology was observed by transmission electron microscopy. hippocampus were cut into 1-mm cubes, fixed with 2.5% glutaraldehyde, and stored at 4°C for 24 h. The cubes were embedded in Spurr’s resin, cut into 0.12-μm-thick sections, and stained with 0.2% lead citrate and 1% uranyl acetate. The images were examined with a transmission electron microscope (JEM-1200X, Shimadzu, Japan) (14).

### Enzyme-linked immunosorbant assay

The hippocampus samples were collected for TNF-α, IL-6, and IL-β detection at 24 h. The concentrations of TNF-α, IL-6, IL-10, and IL β were detected by ELISA kits according to the manufacturer’s instructions. A standard curve was constructed using various dilutions of TNF-α, IL-6, IL-10, and IL-β standard preparation. The levels of the cytokines were calculated according to standard curves.

### Morris water maze

Behavioral tests were conducted on surviving mice 1 week after surgery. Eight mice were taken from the control group and sham group(10 mice in the sepsis group, 2 died at the 7th day), ten mice were taken from the sepsis group(20 mice in the sepsis group, 10 died at the 7th day), fifteen mice were taken from (sepsis + echinomycin) group(20 mice in sepsis group, 5 died at the 7th day), which was used to detect escape latency, target quadrant time and crossing times of mice. MWM was composed of a high 40 cm and a diameter 125cm and a black circular pool, which was divided into four views quadrants. Place a circular platform with a diameter of 10 cm in the center of the third quadrant, For four consecutive days, mice were placed in the pool facing the pool wall, and each mouse entered from four quadrants. Experiment 4 times a day on each mouse with an interval of 20 min and 4 consecutive days intermittent to find the platform. Failed to find the platform of mice in the 60 s, which were guided and allowed to stay on the platform for 20 s. The time the mice spent searching for the platform was recorded as latency. At the 5 th, in the absence of a platform, the mice were placed into the pool wall from the contralateral quadrant (2nd quadrant) of the target quadrant at a fixed time period (9:00 am-12:00 am), and the number of mice crossing the original platform area and the time of staying in the target quadrant are recorded within 60 s.

### Statistical analysis

All statistical comparisons were performed with GraphPad Prism 8.0 Software. Data are presented as the mean ± standard deviation. Repeated measurement and one-way ANOVA were used, and Tukey Kramer, a multiple comparison test, was used for comparison between groups. Kaplan-Meier survival curves for four groups of mice. *p* < 0.05 was considered statistically significant.

## Result

### Echinomycin improved survival rate of CLP mice

**Figure 2:**
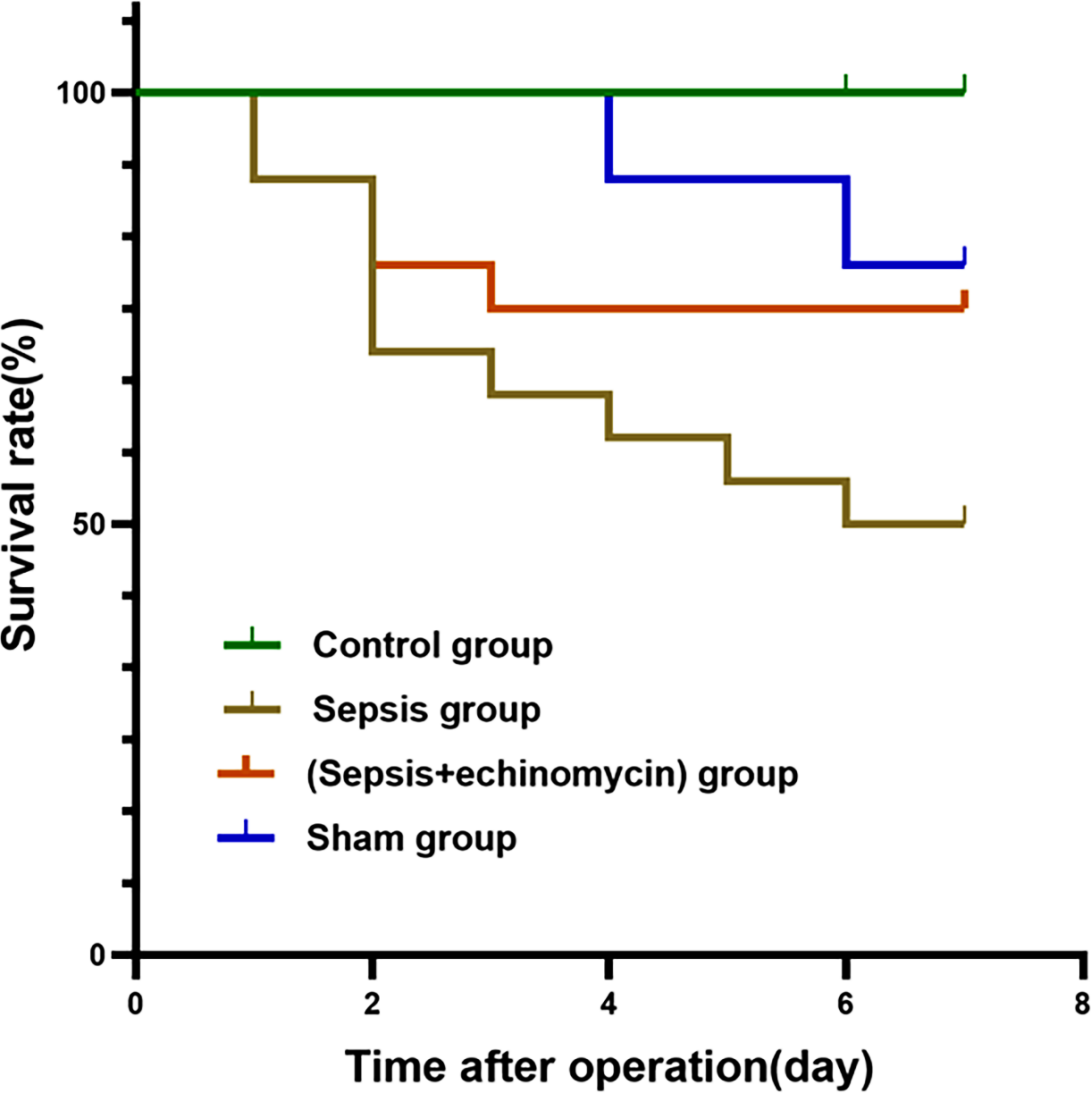
Kaplan-Meier survival curves for four groups of mice. Echinomycin improved survival rate in the first 7 days after CLP.

Mice mortality increased for 7 days after CLP, and survival rate increased with the administration of intraperitoneal echinomycin (75%), compared to the CLP group (50%) (**Figure 2**).

### CLP induces cognitive deficits

**Figure 3:**
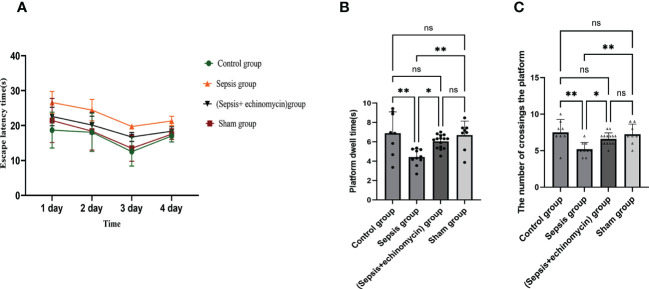
Echinomycin reversed CLP-induced learning and memory impairments. **(A)**:escape latency time. **(B)**:dwell target quadrant time. **(C)**:the number of crossings over the original platform area.*: *p* < 0.05, **: *p* < 0.01, ns:*p* > 0.05.

In the Morris water maze, compared with the control group or sham surgery group, escape latency time increased, and dwell target quadrant time and the number of crossings over the original platform area decreased in the sepsis group. Compared with the sepsis group, escape latency time decreased, and dwell target quadrant time and the number of crossings over the original platform area increased in the (sepsis + echinomycin) group. sepsis induces cognitive deficits, and mice with cognitive deficits improve after administering the HIF-1α inhibitor echinomycin (**Figure 3**).

### CLP induces hippocampus edema

**Figure 4:**
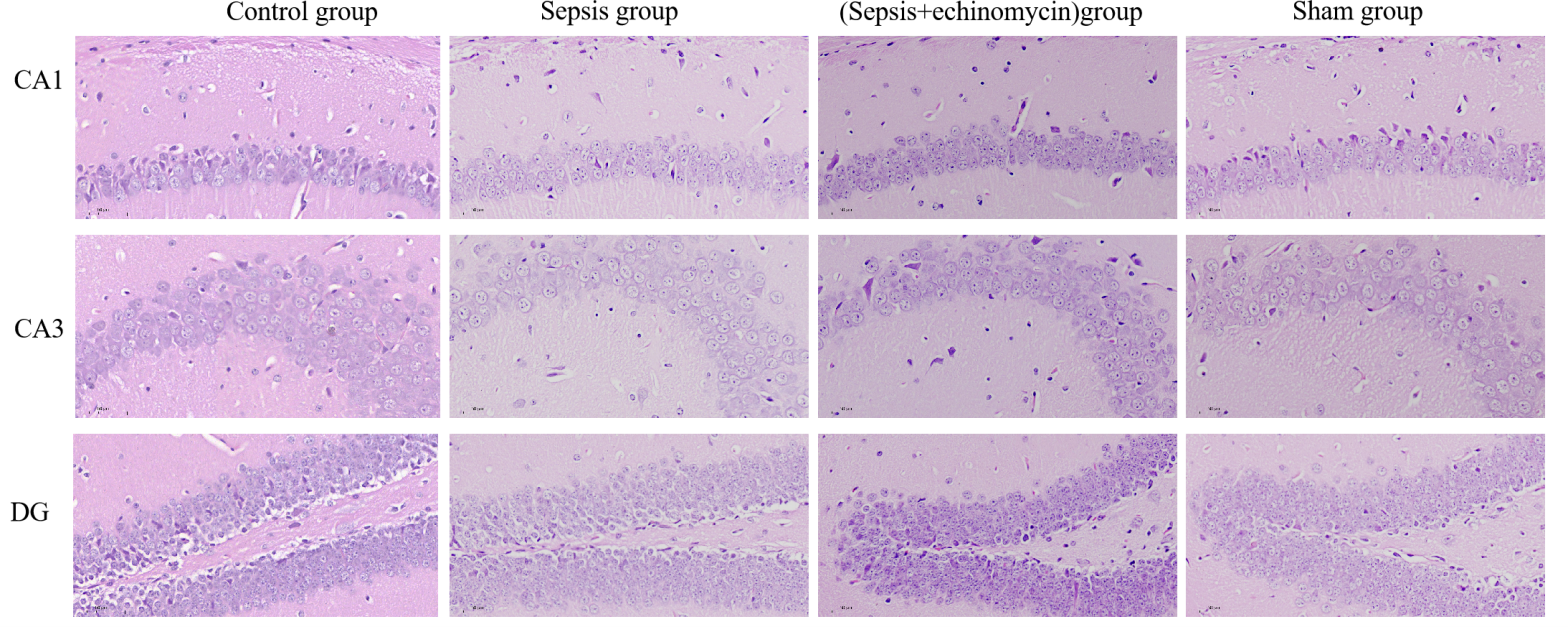
CLP increased CA1,CA3 and DG neuronal damages in the hippocampus of SAE mice. Compared with other three groups, mice in the sepsis group had significant edema of cells, decrease in pyramidal cells, and even lysis in the CA1, CA3, and dentate gyrus (DG) of the hippocampus region 24 h after CLP.

Mice in the sepsis group had significant edema of cells, decrease in pyramidal cells, and even lysis in the CA1, CA3, and dentate gyrus (DG) of the hippocampus region 24 h after CLP. The cells in the sham group and control group were morphologically normal and well-layered. Given the echinomycin group, hippocampus edema was reduced, and pyramidal cells were increased compared to the sepsis group (**Figure 4**).

### CLP up-regulated the HIF-1α and down-regulated BNIP3L protein and m RNA expression in the hippocampus tissue of mice

**Figure 5:**
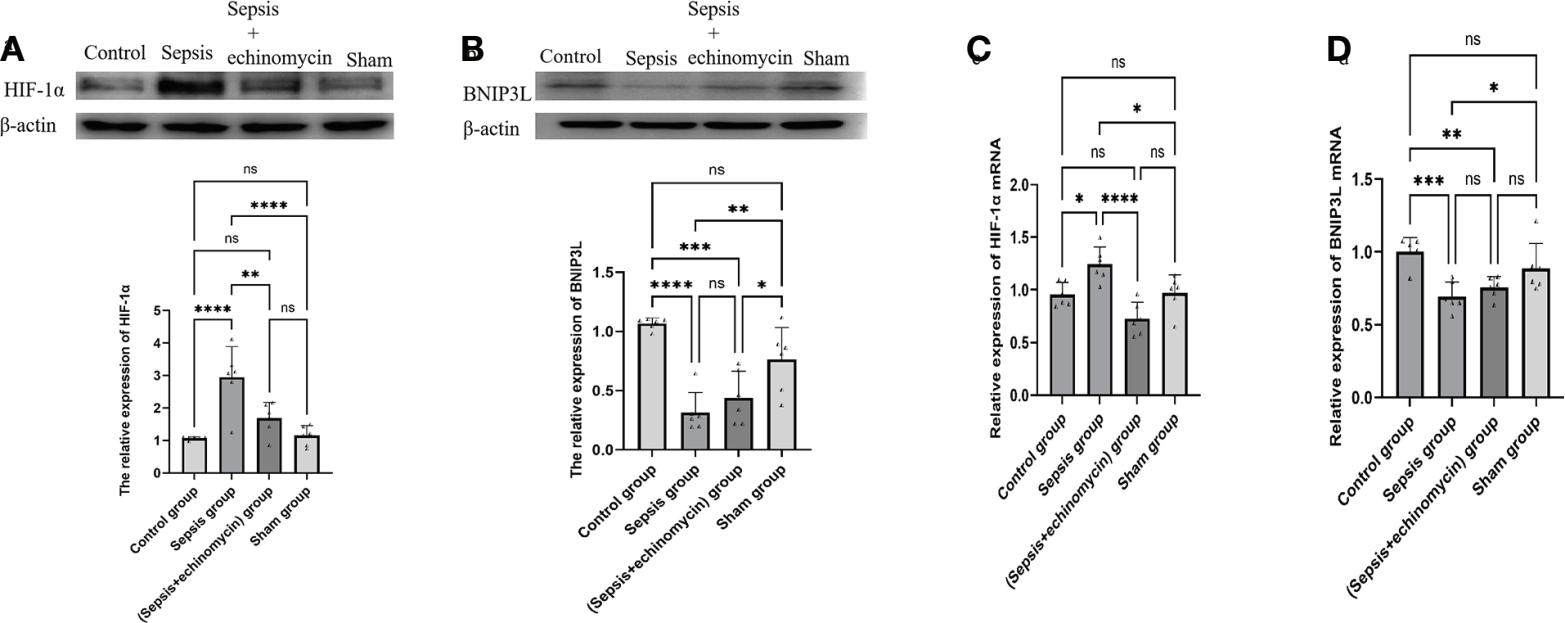
The HIF-1a and BNIP3L protein and m RNA expression in hippocampus tissue. **(A)**: HIF-1α protein expression in hippocampus; **(B)**: BNIP3L protein expression in hippocampus; **(C)**: HIF-1α mRNA expression in hippocampus; **(D)**: BNIP3L mRNA expression in hippocampusCompared with other three groups, sepsis up-regulated the HIF-1a and down-regulated BNIP3L protein and m RNA expression in the hippocampus tissue of mice.*:*p* < 0.05, **:*p* < 0.01, ***:*p* < 0.001,****:*p* < 0.0001, ns:*p* > 0.05.

Compared with the control group and the sham group, the HIF-1α protein (**Figure 5A**) and mRNA (**Figure 5C**) expression of hippocampus was up-regulated, and BNIP3L protein (**Figure 5B**) and mRNA expression(**Figure 5D**) was down-regulated in the sepsis group (*p* < 0.05); after administration of echinomycin to sepsis mice, the HIF-1α protein(**Figure 5A**) and mRNA (**Figure 5C**) was lower than the sepsis group (*p* < 0.05) and the BNIP3L protein (**Figure 5B**) and mRNA (**Figure 5D**) was higher than the sepsis group(*p* > 0.05).

### Immunofluorescence detection the HIF-1α and BNIP3L expression of CA1, CA3 and DG areas of hippocampus

**Figure 6:**
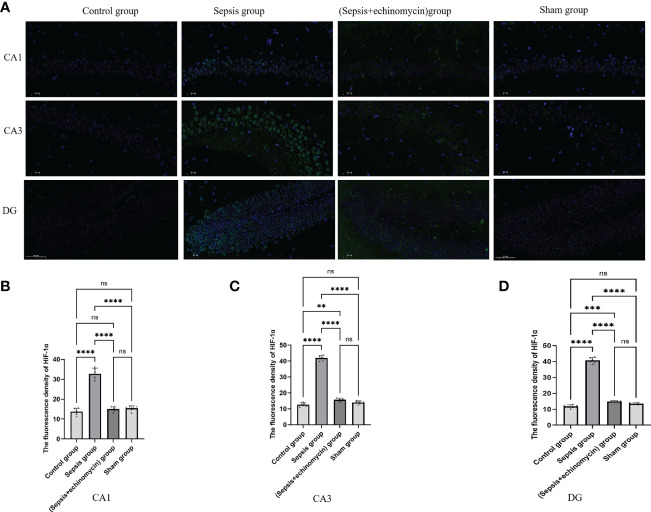
Immunofluorescence detection the HIF-1α expression of hippocampus tissue CA1, CA3 and DG area **(A)**. The average nuclear HIF-1α fluorescence density of hippocampus in three fields of view per slice was analyzed in each group by the ImageJ software **(B–D)**. Compared with other three groups, sepsis up-regulated the HIF-1a expression in the hippocampus of mice.**:*p* < 0.01, ***:*p* < 0.001,****:*p* < 0.0001, ns:*p* > 0.05.

**Figure 7:**
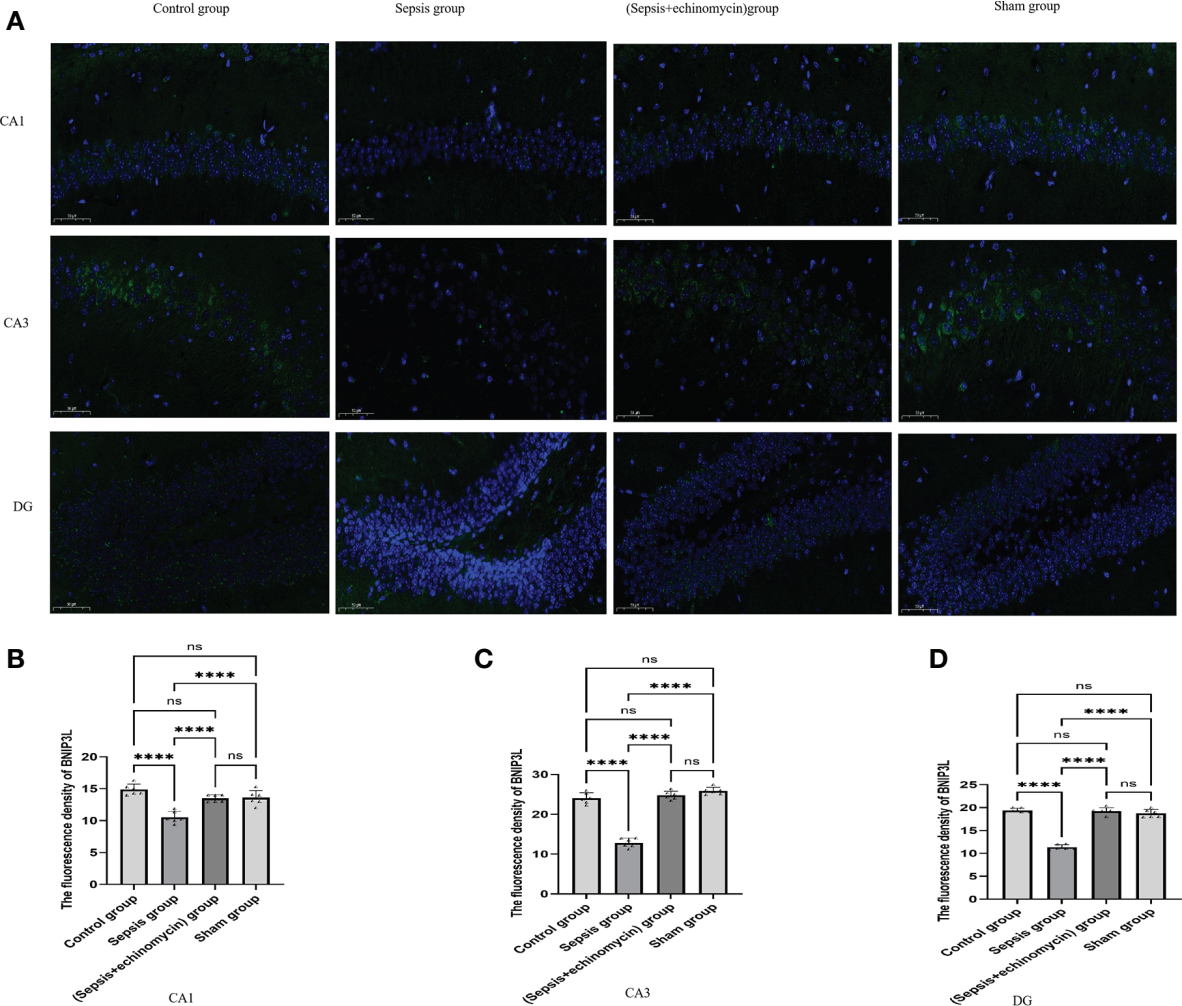
Immunofluorescence detection the BNIP3L expression of hippocampus tissue CA1, CA3 and DG area **(A)**.The average nuclear BNIP3Lfluorescence density of hippocampus in three fields of view per slice was analyzed in each group by the ImageJ software **(B–D)**. Compared with other three groups, sepsis down-regulated BNIP3L expression in the hippocampus of mice.****:*p* < 0.0001, ns:*p* > 0.05.

Immunoassays the expression of HIF1a (**Figure 6**) and BNIP3L (**Figure 7**) in hippocampus CA1, CA3 and DG area. Compared with the control group and the sham group, the number of expressions of HIF-1α increased, and BNIP3L decreased significantly in the sepsis group. Compared with the sepsis group, the number of expressions of HIF 1a decreased, and BNIP3L expression increased after the administration of HIF-1α inhibitors. Compared with the other three groups, the fluorescence density of HIF-1α was significantly enhanced in sepsis group and the fluorescence density of BNIP3 L was significantly reduced in sepsis group(*p* < 0.0001).

### HIF-1α and BNIP3L pathway may induce SAE by promoting apoptosis of hippocampus neurons

**Figure 8:**
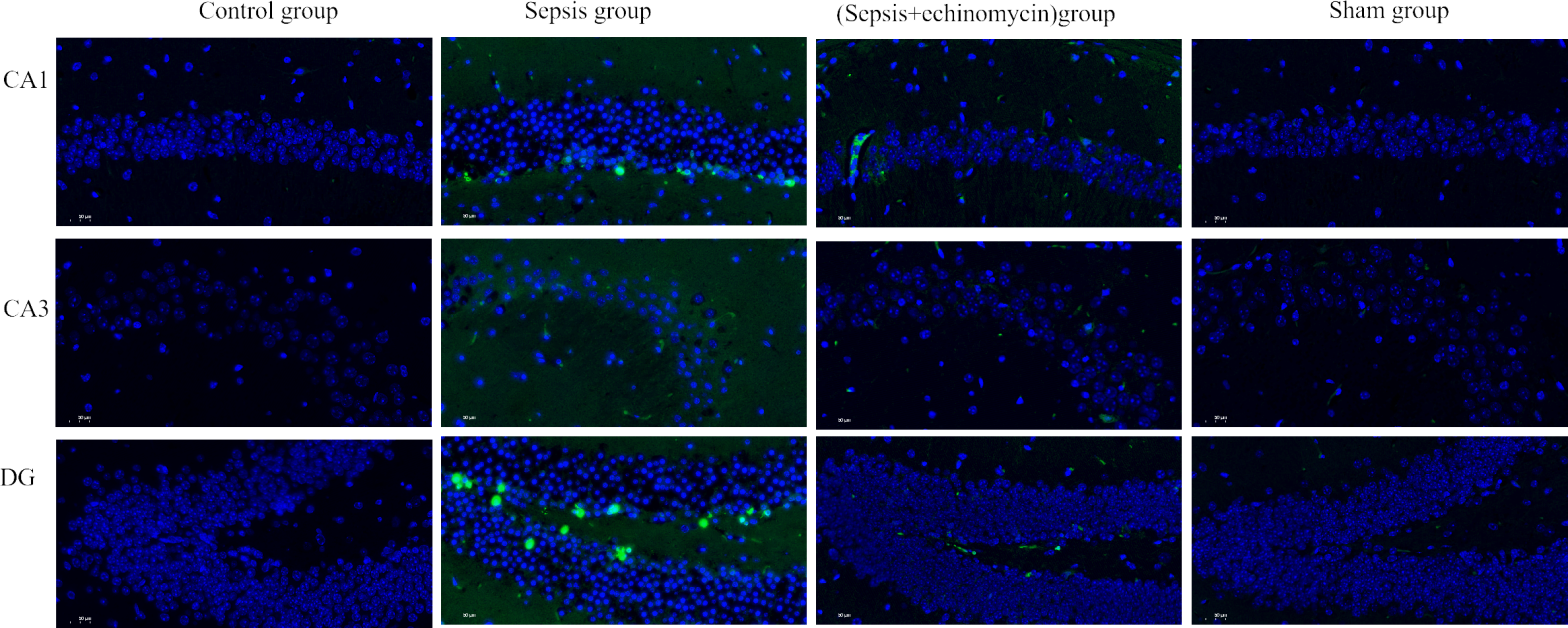
HIF-1α and BNIP3L pathway may induce SAE by promoting apoptosis of hippocampus neurons. Sepsis induce apoptosis of hippocampus neurons, after administration of HIF-1α inhibitor, apoptosis of hippocampus neurons was reduced.

TUNEL staining was used to observe the effect of sepsis on the apoptosis of hippocampus neurons CA1, CA3, and DG area. As shown in the control group and sham group, no hippocampus neurons suffered apoptosis. In the sepsis group, the number of apoptotic hippocampus neurons increased. After echinomycin treatment, the apoptotic neurons of sepsis mice were significantly reduced (**Figure 8**).

### HIF-1α and BNIP3L may induce SAE by promoting mitochondrial damage

**Figure 9:**
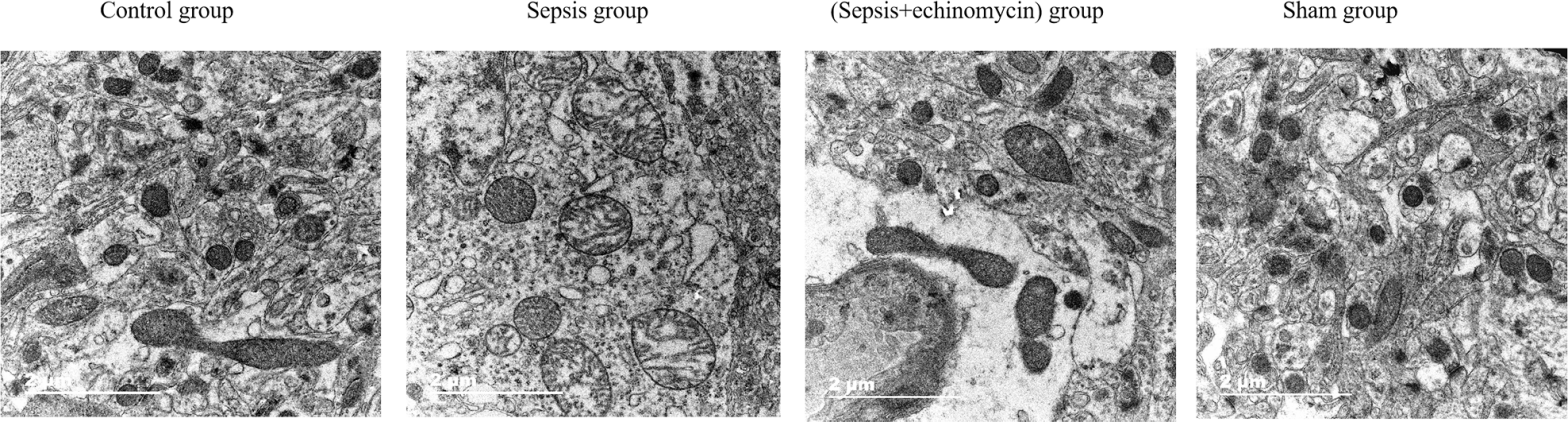
HIF-1α and BNIP3L pathway induced neuronal mitochondrial damage. Compared with other three groups, the mitochondria in the sepsis group underwent obvious swelling, mitochondrial morphological changes, and mitochondrial vacuolization.

In the sham and control groups, the mitochondrial ridges were intact and showed no obvious swelling, whereas the mitochondria in the sepsis group underwent obvious swelling, mitochondrial morphological changes, and mitochondrial vacuolization. After the administration of an echinomycin inhibitor, the swelling of mitochondria was significantly reduced (**Figure 9**).

### The mitochondrial expression of HIF-1α and BNIP3L in sepsis mice

**Figure 10:**
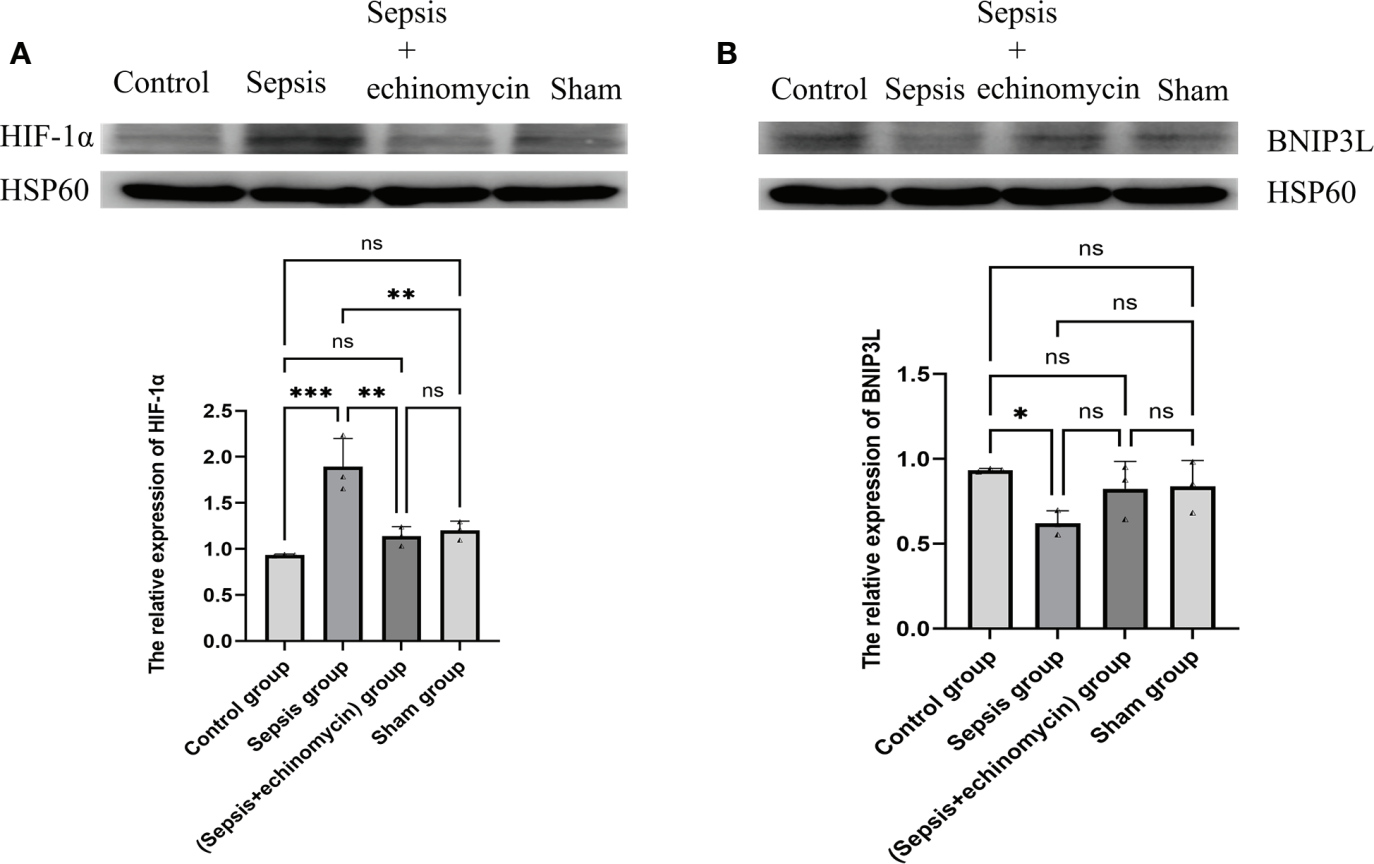
The HIF-1α and BNIP3L protein expression in mitochondrion of hippocampus neuron cell. **(A)**: HIF-1α protein expression in mitochondrion; **(B)**: BNIP3L protein expression in mitochondrion. Compared with other three groups, sepsis up-regulated the HIF-1a and down-regulated BNIP3L protein expression in mitochondrion of hippocampus neuron cell.*: *p* < 0.05, **: *p* < 0.01, ***:*p* < 0.001, ns:*p* > 0.05.

Compared with the control group and the sham group, the HIF-1α protein expression was up-regulated, and the BNIP3L protein expression was down-regulated of mitochondria of the hippocampus in the sepsis group (*p* < 0.05); after administration of echinomycin to sepsis mice, the expression of HIF-1α was down-regulated(*p* < 0.05) and the expression of BNIP3L protein expression was increased(*p* > 0.05) (**Figure 10**).

### HIF-1α and BNIP3L may induce SAE by promoting inflammation in hippocampus

**Figure 11:**
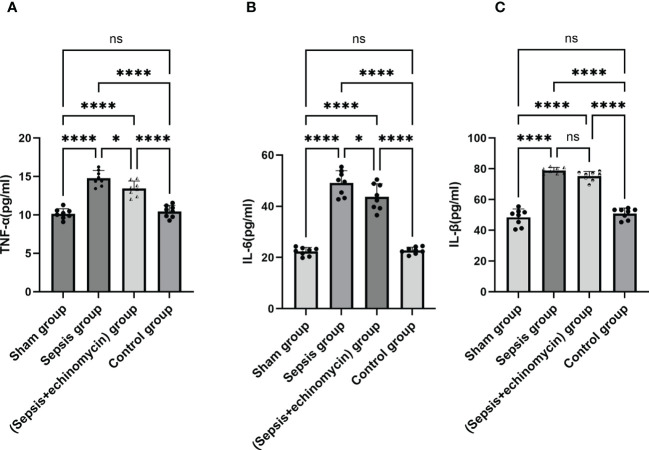
The HIF-1α and BNIP3L pathway induced immune response. **(A)**: TNF-α protein expression in hippocampus; **(B)**: IL-6 protein expression in hippocampus; **(C)**: IL-β protein expression in hippocampus; Sepsis induce the IL-6, IL-β, TNF-α protein expression of hippocampus, TNF-αand IL-6 expression were significantly reduced after administration of HIF-1α inhibitor.*: *p* < 0.05, ****:*p* < 0.0001, ns:*p* > 0.05.

Compared with the control group and the sham group, the IL-6, IL-β, INF-α protein expression of the hippocampus in the sepsis group was up-regulated (*p* < 0.05); After administration of the inhibitor echinomycin, the protein expression of IL-6 and TNF-α were decreased (**Figure 11**).

## Discussion

Many clinical studies confirm that SAE has a high incidence and poor prognosis (2, 3), there are many types of research on the pathogenesis of SAE, but it is still unclear. In this study, it was found that CLP caused hippocampus edema, neuronal apoptosis, mitochondrial damage, and cognitive dysfunction in mice, and after CLP, HIF-1a, IL-6, IL-β, INF-α were highly expressed, and BNIP3L was low expressed in the hippocampus of mice. After administration of the HIF-1α inhibitor, the level of HIF-1a protein and gene expression, IL-6, INF-α were reduced, and the level of BNIP3L protein and gene expression was increased, and the apoptosis damage of hippocampus neurons and mitochondrial damage were alleviated, and cognitive function of sepsis mice and survival rate improved. We speculate that HIF-1α and BNIP3L pathways promote hippocampus neuronal apoptosis, mitochondrial damage, and inflammatory response, which may be the potential mechanism of SAE.

Previous studies have found that HIF 1a and BNIP3L signal pathways play an important role in the pathogenesis of brain diseases such as cerebral ischemia/reperfusion, cerebral hemorrhage, memory ischemia, glioma and Alzheimer’s disease.The expression levels of HIF-1α and BNIP3L are increased. Intervention of the expression levels of HIF-1α and BNIP3L can protect brain injury (15–17). In this study, we found abnormal expression of HIF 1a, BNIP3L protein, and gene in the hippocampus and mitochondrion of sepsis. The hippocampus structures and mitochondrial structures were obviously damaged. After the HIF 1a inhibitor was used to inhibit the expression of HIF 1a and increase the expression of BNIP3L, the damage to hippocampus structures and mitochondrial structure was significantly reduced, and the cognitive function of sepsis mice were significantly improved. The above research shows that the HIF 1a, BNIP3L pathway may also be the potential mechanism of SAE.

Snyder B et al. found that the expression of HIF-1a in the hippocampus of aged mice significantly increased after hypoxia, which promoted hippocampus injury and induced memory dysfunction (18). The research of Qiang Zhang et al. found that astragalus extract decreased the expression of HIF-1a at both the mRNA and protein levels in the hippocampus and reduced the apoptosis of hippocampus neurons (19). Xin Rui Zhao et al. found in the study of the uterosacral ligament that the surface of HIF-1α increased, BNIP3L increased, apoptosis increased, cell viability decreased, and the expression of apoptosis-related proteins increased (20). The above studies suggest that HIF-1α is an important molecule that causes central nervous system injury and apoptosis. In this study, we found that the expression of HIF-1α in the hippocampus of sepsis mice increased, the hippocampus structure was damaged, hippocampus neurons were apoptosis, and the mice developed cognitive dysfunction. After the inhibition ofHIF-1α, the results of the damaged hippocampus, neuronal apoptosis, and cognitive function of mice were significantly improved. Our research results are consistent with previous studies. Besides, in this study, we explored the relationship between the HIF 1a pathway and inflammatory factors TNF a, IL-6, and IL-β. We found that after the administration of HIF 1a, inflammatory factors TNF a and IL-6 decreased compared with the sepsis group. We speculate that HIF-1α promotes hippocampus injury, neuronal apoptosis, mitochondrial damage, and inflammatory reaction may be the mechanism of SAE.

Mitochondrial autophagy is a double-edged sword. In some cases, dysfunctional mitochondrial autophagy can lead to nervous system injury, including excessive mitochondrial autophagy and autophagy defects (21–23). BNIP3L plays an important role in mitochondrial autophagy and in maintaining the normal morphology and function of mitochondria (24, 25). Fu ZJ et al. found that HIF -1α in renal tubular cells BNIP3L mediated mitochondrial autophagy induces mitochondrial damage and damages the kidney (26). Yan Zhang et al. found that myocardial injury increased the expression level ofHIF-1α, activated the downstream BNIP3L and subsequently triggered mitochondria-dependent autophagy (27). Zhang H et al. and the above research confirmed that HIF 1a and BNIP3L play an important role in mitochondrial autophagy (28). Yang Yuan et al. found that Bnip3l knockout (bnip3l) impaired mitophagy and cultivated cerebra I-R injury in mice, which can be recovered by BNIP3L expression (24). Ria ling Ma et al. found that BNIP3L expression decreased after traumatic brain injury and colocalized with neuronal cells in cortical areas. Upregulation of BNIP3Lexpression and autophagy was increased, while neuronal apoptosis and brain water content decreased along with neurological deficits (25). The above studies found that the expression of BNIP3L was reduced, and the mitochondrial autophagy was defective, promoting neuronal apoptosis, which plays an important role in central nervous system diseases. Our study results showed that the expression level of BNIP3L in the hippocampus and mitochondria of mice were reduced after sepsis. After administration of the HIF-1α inhibitor, the expression of BNIP3L was up-regulated, and neuron apoptosis and mitochondrial damage were significantly improved. Therefore, the decrease in BNIP3L expression level may play an important role in SAE.

Previous studies found that HIF-1α can activate the expression of BNIP3L in other diseases (8, 29). However, in this study, we found that HIF-1α did not activate BNIP3L expression. After the HIF-1α inhibitor was given, the expression of BNIP3L increased. There may be other potential mechanisms regulating BNIP3L expression in sepsis, which may be attributed to ubiquitination. The increase of HIF-1α expression in a hypoxia environment will promote the increase of polyubiquitination activity. The increase of ubiquitination may lead to the degradation of BNIP3L. When the HIF-1α inhibitor is given, the ubiquitination activity will decrease, leading to the reduction of BNIP3L ubiquitination degradation (22, 30).

Several limitations must be clarified in this study. In this study, we only explored the relationship between HIF 1a and BNIP3L pathways and sepsis mice in the acute phase, but the long-term relationship with sepsis mice is still unclear. The mechanism of HIF 1a and BNIP3L pathway leading to apoptosis of hippocampus neurons and mitochondrial damage is still unclear. We only observed morphological changes and need further experimental research to explore.In this study, only HIF-1α inhibitors were given, but the expression level of BNIP3L was not directly interfered, which may lead to deviation in the results of this study.

## Conclusion

This study is to explore the relationship between HIF-1α, BNIP3L pathway, and SAE mice. The relationship between HIF-1α, BNIP3L pathway and mice behavior, the morphology of hippocampus, hippocampus neurons, mitochondria, and inflammatory response. We speculate that HIF-1α, BNIP3L pathway mediates apoptosis of hippocampus neurons, mitochondrial damage, and inflammatory response may be the mechanism of SAE. The intervention of HIF-1α, BNIP3L pathway expression may be could improve the cognitive dysfunction of SAE mice.

## Data availability statement

The original contributions presented in the study are included in the article/supplementary files. Further inquiries can be directed to the corresponding authors.

## Ethics statement

The animal study was reviewed and approved by Ethical Use of Animals of Tianjin Medical University General Hospital.

## Author contributions

LZ, YL and KX design the study, analyzed, and draft the article. LZ and YL performed the majority of the experiments. YSo, YZ, HL, YSh and YF assisted with tissue sample collection. LZ and YL analyzed the data. LZ wrote the first draft of the manuscript, YL and KX revised the paper, worked on the English, and made the final version. All authors contributed to the article and approved the submitted version.
